# Deciphering the Kidney Matrisome: Identification and Quantification of Renal Extracellular Matrix Proteins in Healthy Mice

**DOI:** 10.3390/ijms24032827

**Published:** 2023-02-01

**Authors:** Umut Rende, Seong Beom Ahn, Subash Adhikari, Edward S. X. Moh, Carol A. Pollock, Sonia Saad, Anna Guller

**Affiliations:** 1 ARC Centre of Excellence in Nanoscale Biophotonics, The Graduate School of Biomedical Engineering, University of New South Wales, Sydney, NSW 2052, Australia; 2 Macquarie Medical School, Macquarie University, Macquarie Park, NSW 2109, Australia; 3 Advanced Technology and Biology Division, The Walter and Eliza Hall Institute of Medical Research, Melbourne, VIC 3052, Australia; 4 Department of Medical Biology, University of Melbourne, Melbourne, VIC 3052, Australia; 5 ARC Centre of Excellence for Nanoscale BioPhotonics, Macquarie University, Sydney, NSW 2109, Australia; 6 Department of Medicine, Kolling Institute of Medical Research, University of Sydney, St. Leonards, NSW 2065, Australia

**Keywords:** extracellular matrix, matrisome, kidneys, proteomics, mass spectrometry, mouse, tissue extraction, protein identification, label-free quantification (LFQ) of proteins

## Abstract

Precise characterization of a tissue’s extracellular matrix (ECM) protein composition (matrisome) is essential for biomedicine. However, ECM protein extraction that requires organ-specific optimization is still a major limiting factor in matrisome studies. In particular, the matrisome of mouse kidneys is still understudied, despite mouse models being crucial for renal research. Here, we comprehensively characterized the matrisome of kidneys in healthy C57BL/6 mice using two ECM extraction methods in combination with liquid chromatography tandem mass spectrometry (LC-MS/MS), protein identification, and label-free quantification (LFQ) using MaxQuant. We identified 113 matrisome proteins, including 22 proteins that have not been previously listed in the Matrisome Database. Depending on the extraction approach, the core matrisome (structural proteins) comprised 45% or 73% of kidney ECM proteins, and was dominated by glycoproteins, followed by collagens and proteoglycans. Among matrisome-associated proteins, ECM regulators had the highest LFQ intensities, followed by ECM-affiliated proteins and secreted factors. The identified kidney ECM proteins were primarily involved in cellular, developmental and metabolic processes, as well as in molecular binding and regulation of catalytic and structural molecules’ activity. We also performed in silico comparative analysis of the kidney matrisome composition in humans and mice based on publicly available data. These results contribute to the first reference database for the mouse renal matrisome.

## 1. Introduction

The extracellular matrix (ECM) is a complex macromolecular network that surrounds the cells of all tissues and organs [1]. The ECM is a product of the cells; thus, it is organ- and tissue-specific. By providing adhesion and anchorage to the cells [2], the ECM ensures tissue integrity. The composition and architecture of the ECM change with modifications of cellular phenotypes. In turn, the differentiation and biological activity of the cells are reciprocally controlled via signaling from the ECM [1,3,4]. Proteins of the ECM (collectively termed the “matrisome” [5]) play an essential role in tissue and organ development [6] and cellular metabolism [7]. According to the classification proposed by Naba et al. [5], the matrisome consists of the “core matrisome” and “matrisome-associated proteins”. The core matrisome is formed by structural ECM components (collagens, ECM glycoproteins and proteoglycans) [8,9]. The term “matrisome-associated proteins” covers the proteins which are found in the ECM but can not be classified as core matrisome components. These proteins are further categorized as (i) ECM-affiliated proteins, which have a similar architecture to ECM proteins and/or are known to be associated with ECM proteins, (ii) ECM regulators, and (iii) secreted factors, which are shown to interact with the ECM [5,10]. Identified matrisome proteins of different human and mouse tissues and tumors are deposited in the “Matrisome Database” (MD) (http://matrisomeproject.mit.edu/, accessed on 23 January 2023).

Pathological changes of the matrisome (e.g., excessive accumulation, destruction or dysfunctionality of certain proteins or protein groups) orchestrate many diseases of diverse etiologies, with particular involvement in inflammation, wound healing, cancers and fibrosis [11,12,13]. A deep analysis of the matrisome is an invaluable instrument for understanding tissues’ and organs’ functions, as well as the mechanisms and biomarkers of various diseases.

In the last decade, mass spectrometry (MS) has become the leading analytical approach in matrisome studies. It is intensively used for the discovery of biomarkers and assessment of the ECM [14]. Rapid technological developments in proteomics, such as enhanced sample preparation protocols, database searching, and bioinformatics analysis now allow unbiased protein quantification, identification and characterization, including the discovery of post-translational modifications (PTMs) [15]. However, despite these impressive advancements, standard protein extraction techniques, which use whole tissue lysates, need to be better adapted to matrisome studies. The continuing challenges in ECM proteomics are the enrichment of matrisome proteins in the samples by separating low-abundance ECM proteins from intracellular proteins, and solubilizing heavily cross-linked [9] extracted proteins. Additionally, identification and quantification of the matrisome proteins in the presence of abundant PTMs is a complex task [16].

To tackle the difficulty of matrisome enrichment, several variations of decellularization methodologies that gradually reduce the amount of cellular components in the sample while preserving the ECM proteins have been proposed. The most widely used extraction method applicable for proteomic studies of the matrisome [5,17,18,19,20] employs a commercially available kit from Millipore for tissue protein fractionation by sequential incubations of the sample in buffers of different pH, salt and detergent concentrations. This procedure results in the biochemical separation and removal of the proteins of the cells’ cytosolic, nuclear, membrane and cytoskeletal compartments. It then allows the enrichment of ECM proteins in the residual insoluble product. This method is further referred to as “Compartmental Matrix Enrichment” (CME). Another method is termed “Sequential Matrix Enrichment” (SME). It uses guanidine hydrochloride (Gu-HCl) to further solubilize insoluble matrisome proteins after a decellularization step [20,21,22,23]. This extraction method is performed with an ionic (NaCl) buffer to additionally extract loosely bound ECM proteins (enzymes, secreted factors, ECM-associated and newly deposited proteins), as well as a low-concentration detergent (sodium dodecyl sulphate, SDS) with a shorter incubation time to eliminate intracellular proteins, and then uses a Gu-HCl buffer to enhance the obtaining of heavily cross-linked ECM proteins.

**Figure 1:**
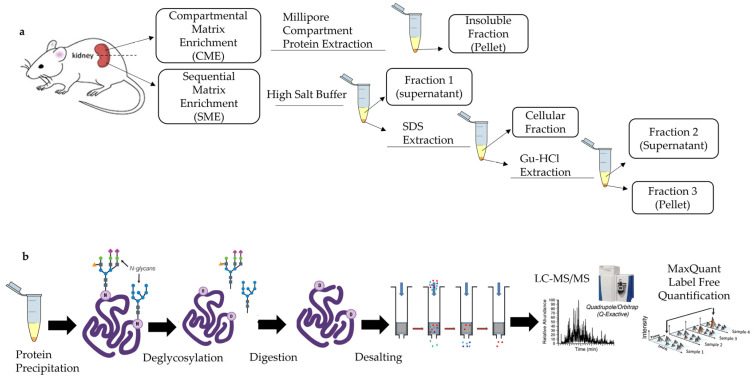
Schematic illustration of the methods applied in the current study. (**a**) Matrisome protein extraction from healthy mouse kidneys by CME and SME methods. (**b**) Further processing and analysis of proteins after obtaining samples from methods CME and SME. Yellow color in the extraction products indicates the supernatant, and the orange color shows insoluble pellets. Abbreviations: SDS (Sodium Dodecyl Sulfate) and Gu-HCl (Guanidine Hydrochloride).

In the current study, we applied these two ECM extraction methods (Figure 1a) to explore the matrisome of kidneys in healthy adult mice with an overall goal of creating a reference database for future renal research. This work was particularly motivated by the fact that, now, information on the matrisome composition of mouse kidneys is very limited and incomplete. While mouse models are crucial for studies of renal morphogenesis and diseases [24], chronic kidney disease (CKD) [25], as well as for drug and biomarker discovery [26,27], only two recent studies are currently available on the identification of ECM proteins in the insoluble tissue fractions of mouse kidney extracts [20,28]. Importantly, the methodology applied in these studies did not allow the identification of loosely bound ECM proteins, which suggests incomplete characterization of the matrisome. Moreover, quantification of ECM proteins in mouse kidneys has yet to be achieved.

The matrisome proteins extracted using CME and SME methods were further studied with liquid chromatography using a tandem mass spectrometry (LC-MS/MS) system (Figure 1b). Firstly, the identified proteins were explored with the Matrisome Database (MD) and UniProt databases. To compare the abundance of identified matrisome proteins from the two extraction methods, MaxQuant label-free quantification (LFQ) was employed, and the data was then analyzed using LFQ-Analyst [29].

## 2. Results

### 2.1. Qualitative Analysis of Mouse Kidney Matrisome

#### 2.1.1. Identification of the Proteins of the Mouse Kidney Matrisome

The raw data results of the proteomic study are available in Table S1 in Supplementary Materials.

**Figure 2:**
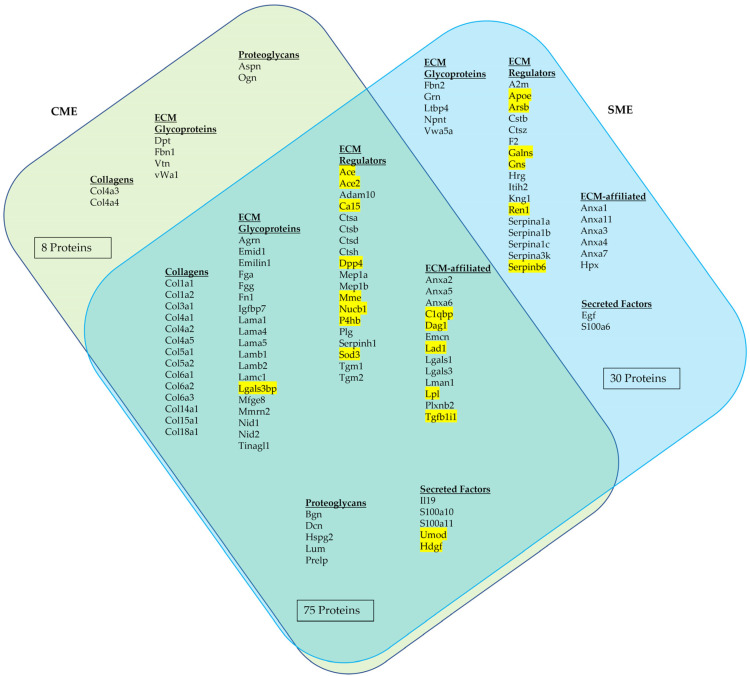
Identified matrisome proteins with unique peptides obtained from the samples processed via CME and SME methods. Venn diagram illustrates the common and unique matrisome proteins identified using each method. The proteins highlighted proteins in yellow have not been previously listed in the MD.

Based on unique peptides, a total of 2442 proteins were identified by the CME and SME methods. Collectively, this resulted in the identification of a total of 113 matrisome proteins in healthy mouse kidneys. The summary on the mouse kidney matrisome composition identified in the current study is shown in Figure 2. The detailed and referenced lists of the matrisome proteins identified in the current study by using CME and SME approaches and classified by the MD divisions and categories are presented in Table A1 (collagens), Table A2 (ECM glycoproteins), Table A3 (ECM proteoglycans), Table A4 (ECM regulators), Table A5 (ECM-affiliated proteins), and Table A6 (ECM secreted factors), in Appendix A.

As follows from Figure 2, among the proteins identified by combination of the two ECM extraction and enrichment methods, 51 (45%) were classified as core matrisome proteins. By frequency of observation, the core matrisome of healthy mouse kidneys was dominated by ECM glycoproteins (28 identified proteins), followed by sixteen collagens and seven ECM proteoglycans. The remaining 62 identified proteins (55%) were regarded as matrisome-associated proteins. Most of the identified matrisome-associated proteins belonged to the category of ECM regulators (36 proteins). This division of the matrisome also included nineteen ECM-affiliated proteins and seven secreted factors.

Notably, 22 proteins among the 113 identified ECM proteins have not been previously classified in the MD (see Figure 2, the yellow highlights; refer to Table A7, Appendix A for the more detailed descriptions). These 22 proteins were attributed as matrisome components based on their extracellular location, functions, and, also, on their interactions with extracellular proteins—as reported in the literature and in the UniProt database. Then, they were classified into the Matrisome categories of ECM glycoproteins, ECM regulators, ECM-affiliated proteins, and secreted factors. The majority of the newly classified mouse kidney matrisome proteins belonged to the division of the matrisome-associated proteins, including 14 ECM regulators, five ECM-affiliated proteins, and two secreted factors, and only one was classified as a core matrisome component (an ECM glycoprotein: Galectin-3-binding protein). Notably, six of these matrisome proteins identified in mouse kidneys for the first time were revealed only by SME.

#### 2.1.2. Gene Ontology Analysis of Identified Mouse Matrisome Proteins

**Figure 3:**
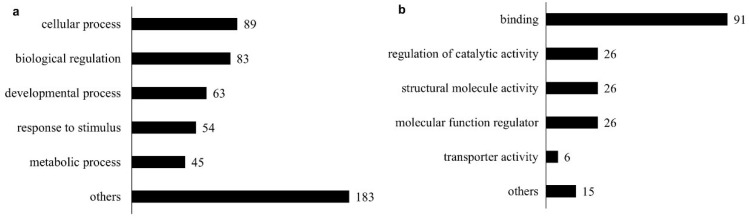
Mapping of the identified healthy mouse kidney matrisome proteins against GO terms using Uniprot database: (**a**) GO terms for the Biological Process and (**b**) GO terms for the Molecular Function.

Gene Ontology (GO)-enriched terms analysis based on the top five biological processes revealed that the majority of the identified mouse kidney matrisome proteins primarily belonged to the classes of “cellular process” (GO:0009987), “developmental process” (GO:0032502), “response to stimulus” (GO:0048583) and “metabolic processes” (GO:0019538) and other processes, including multicellular organismal processes, biological adhesion localization, immune system processes, locomotion, interspecies biological processes, reproductive processes, behavior, biomineral tissue development, growth, viral processes, trans-synaptic signaling, removal of superoxide radicals and estrous cycle (Figure 3a). These attributions indicate a key involvement of the matrisome in the regulation of the cellular physiological activity in the kidneys.

The top five GO-enriched terms for molecular function of identified proteins revealed the “binding (GO:0005515)” proteins, which interact with other molecules via a specific site, as a leading category. This was followed by “regulation of catalytic activity (GO:0050790)”, “structural molecule activity” (GO:0005198), “molecular function regulator” (GO:0098772), “transporter activity” (GO:0005215) and others including signaling receptor activity, transcription coregulators, scavenger receptor activity, antioxidant activity, ATPase activity, translation activator activity and protein–macromolecule adaptors (Figure 3b).

#### 2.1.3. Comparative Efficiency of ECM Extraction Methods in Mouse Kidneys Matrisome Identification

Table A8 in Appendix A shows the outcomes of the CME and SME methods in detection of matrisome composition in terms of the number of proteins belonging to various categories of the matrisome. As follows from Figure 2 and detailed in Table A8, the SME method allowed identification of more matrisome proteins than CME (105 vs. 83, respectively).

There were no statistically significant differences in identification of proteins belonging to matrisome divisions and categories compared to the total number of proteins revealed by each method individually or both methods in total. However, in comparing the 113 mouse kidney matrisome proteins identified in total in the current study to the combination of two ECM extraction methods, SME contributed to the observed outcomes more than CME (105/113, or 93% vs. 83/113, or 73%, with the CI_95%_ [87; 99]% vs. [64; 82]%, respectively). The relative efficiency of CME and SME in the detection of core matrisome proteins was statistically similar (41%, CI_95%_ [31; 51]% vs. 38% CI_95%_ [28; 48]%, respectively). At the same time, SME identified more matrisome-associated proteins than CME in the totally detected matrisome (62/113, or 55%, CI_95%_ [45; 65]% vs. 37/113, or 33%, CI_95%_ [23; 42]%, respectively). Both of the ECM enrichment methods used in this study demonstrated similar efficiency in identification of the proteins belonging to the subordinal categories of the matrisome.

#### 2.1.4. Comparative Analysis of the Composition of the Mouse and Human Kidney Matrisome

**Figure A1:**
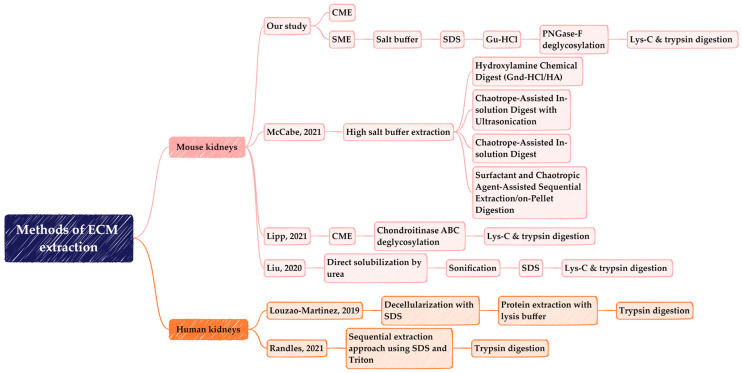
Comparison of the methods of extraction of ECM proteins from mouse and human kidneys tissues.

Next, we compared our results regarding matrisome protein identification with the published data on the healthy mouse kidney ECM proteins identified in recent studies [20,28,30] and the published data on the matrisome proteins from healthy human kidneys [31,32]. It is important to note that across the studies included in this comparison, different protein extraction and analysis methods were used (Figure A1 in Appendix A). There was also a variation in the sample size, age and gender of mice and humans (Table A9 in Appendix A).

By combining our data with other published data obtained in mouse kidney matrisome studies [20,28,30], in total 229 different proteins have been identified. Prior studies [31,32] together identified 178 matrisome proteins in adult human kidneys. The comparison of mouse and human data revealed that 134 matrisome proteins are shared between mouse and human matrisomes, while 95 of these proteins were found only in mouse kidneys and 44 were identified only in human kidneys (Table A10, Table A11, Table A12, Table A13, Table A14 and Table A15 in Appendix A).

**Figure A2:**
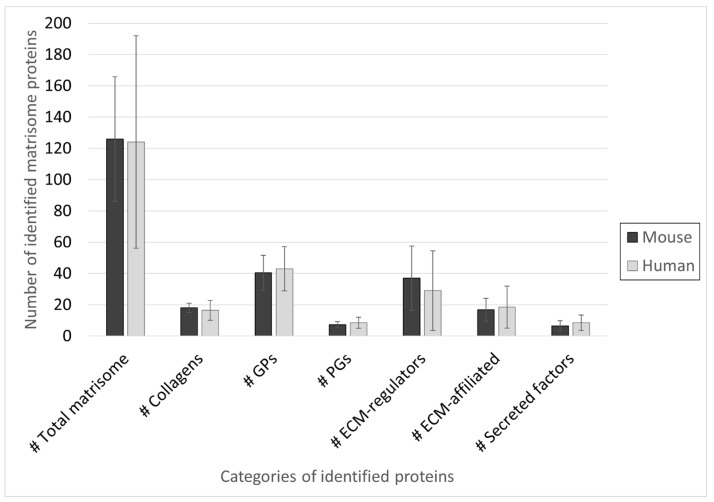
The average composition of the matrisome revealed in mouse and human kidney samples by different studies, including the current study, and the following publications by McCabe et al. [20], Lipp et al. [28], Liu et al. [30], Louzao-Martinez et al. [31], and Randles et al. [32]. The bar graphs show Mean ± Standard Deviation of the number of proteins in each category averaged across the species-specific data reported in Table A9. Abbreviations: #—number of; GPs—ECM Glycoproteins; PGs—Proteoglycans.

A summary of the contribution of the identified proteins of different categories to mouse and human matrisomes based on the data reported in the current study and the cited references is shown in Figure A2 in Appendix A. This figure indicates that the qualitative composition of mouse and human matrisome is similar. The average numbers of identified matrisome proteins in mouse and human kidneys are 126 and 124, respectively. A similar number of proteins were identified in the matrisome categories as well (eighteen and seventeen collagens, forty-one and forty-three ECM glycoproteins, seven and nine proteoglycans, thirty-seven and twenty-nine ECM regulators, seventeen and nineteen ECM-affiliated proteins, and seven and nine secreted factors, respectively).

### 2.2. Mouse Kidney Matrisome Protein Quantification

**Figure 4:**
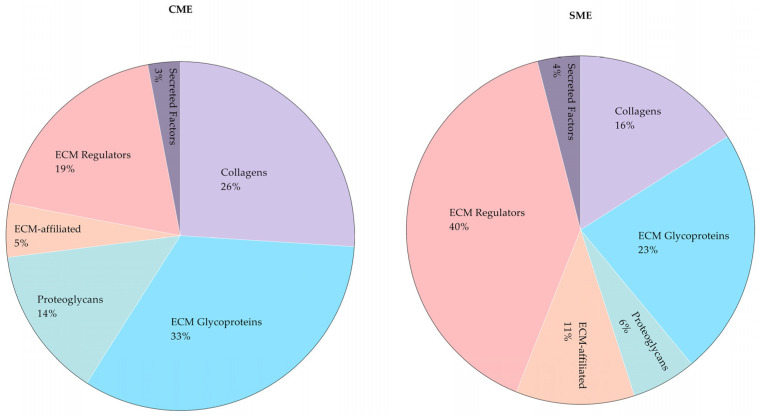
Relative abundance of matrisome proteins in healthy mouse kidneys according to MaxQuant LFQ protein intensities. The results are presented separately for the products of CME and SME methods.

The quantitative characterization of the healthy mouse kidney matrisome was performed by analyzing the MaxQuant LFQ protein intensities data. The analysis revealed a total of 87 distinct matrisome proteins that were able to be quantitatively examined (Figure 4).

From the total LFQ intensities, the core matrisome of the healthy mouse kidneys is predominantly composed of ECM glycoproteins (45% or 51%, as quantified following the CME and SME sample preparation methods, respectively). Collagens comprise 36% of the core mouse kidney matrisome according to both extraction methods. Proteoglycans form 19% or 13% of the core matrisome, according to the quantifications of the samples prepared by CME and SME, respectively. The matrisome-associated proteins in mouse kidneys are represented mostly by the ECM regulators, followed by the ECM-affiliated proteins and secreted factors.

**Figure 5:**
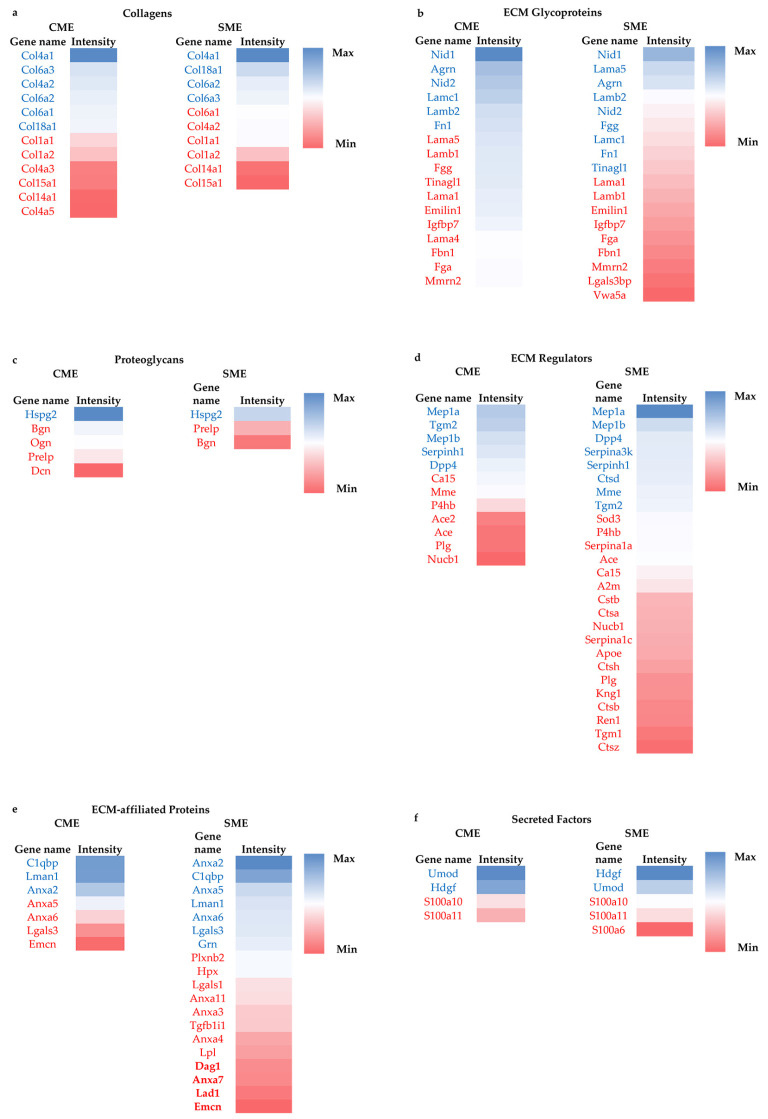
Heatmap of abundance of quantifiable matrisome proteins in the products of the CME and SME methods of sample preparation. Color coding in the heatmap depicts the variation between the maximum (coded in blue tones) to minimum (coded in red tones) observed LFQ intensity for each matrisome category and protein extraction method. The protein names shown by blue and red fonts are expressed above and below the average LFQ values in each matrisome category, respectively. The color coding scales (from blue for maximum LFQ intensity to red for minimum intensity per category) are provided on side of heatmaps (**a**–**f**).

The detailed presentation of the results of quantitative analysis of the mouse kidney matrisome is visualized in Figure 5. As can be seen from this figure, using both ECM enrichment methods, collagen type IV (Col4a1) is defined as the most abundant collagen, with collagen type VI (Col6a1, Col6a2, Col6a3) and collagen type XVIII (Col18a1) also being expressed at above the average level of LFQ intensities observed for collagens. The most highly expressed ECM glycoproteins quantified in the products of both CME and SME include nidogen (Nid1, Nid 2), agrin (Agrn), laminin (Lama5, Lamb2, Lamc1), and fibronectin (Fn1). The top-expressed proteoglycan is perlecan (Hspg2). Among ECM regulators, the top-expressed proteins include meprin A (Mep1a, Mep1b), protein-glutamine gamma-glutamyltransferase 2 (Tgm2), serpin H1 (Serpinh1), and Dipeptidyl peptidase 4 (Dpp4). The category of ECM-affiliated proteins is dominated by the complement component 1 Q subcomponent-binding protein (C1qbp), protein ERGIC-53 (Lman1), and annexin A2 (Anxa2; plus, Anxa5 and Anxa6 that were only overexpressed in the product of SME). Overexpressed matrisome-associated secreted factors included uromodulin (Umod) and hepatoma-derived growth factor (Hdgf).

Some of the quantifiable proteins were more abundant either in CME or SME, while other proteins were detected in similar intensities in the products of both extraction methods. In general, CME allowed quantification of more intensities of core matrisome proteins, but fewer ECM-associated proteins, compared to SME. On the other hand, SME processing detected a higher abundance of ECM-affiliated proteins, ECM regulators and secreted factors than did CME. The complete list of protein FC data between SME fractions and CME is presented in Table S2 in the Supplementary Information.

**Figure A3:**
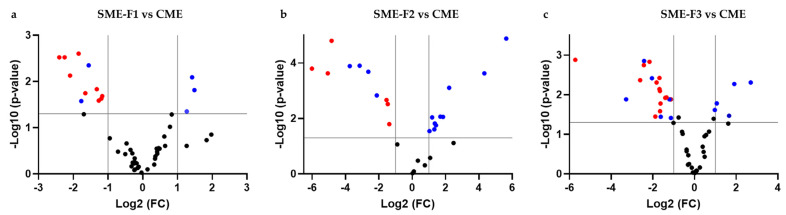
Quantitative comparison of protein extraction levels between SME fractions and CME. Volcano plots represent significant differences in protein abundances (FC > 2, *p*-value < 0.05) between (**a**) SME (fraction 1) and CME, (**b**) SME (fraction 2) and CME and (**c**) SME (fraction 3) and CME. Top-right-hand sides of volcano plots are proteins that were significantly higher in SME fractions. Top-left-hand sides are proteins that were significantly higher in CME. Blue dots indicate Matrisome-associated proteins and red dots indicate core matrisome proteins that were significantly higher in either SME fractions or CME.

The comparative analysis of the LFQ quantification of ECM proteins extracted by CME and SME methods also revealed the following. Fifty one matrisome proteins (ten collagens, sixteen ECM glycoproteins, three proteoglycans, eleven ECM regulators, seven ECM-affiliated and four secreted factors) were shared between the products of both extraction methods. CME additionally allowed quantification of six matrisome proteins (two collagens, one ECM glycoprotein, two proteoglycans and one ECM regulator). SME added another 30 quantifiable matrisome proteins to the list (two ECM glycoprotein, fifteen ECM regulator and twelve ECM-affiliated and one secreted Factor). The comparative analysis of the quantification efficiency of the two studied protein extraction methods shows that there were no significant differences in abundances of shared matrisome proteins. However, higher FCs were observed in the majority of the proteins in CME, with the exception of one collagen (Col14a1), four ECM regulators (Ace, P4hb, Plg and Nucb1), four ECM-affiliated (Anxa2, Anxa5, Anxa6 and Lgals3) and two secreted factors (S100a10 and S100a11) (Figure A3 in Appendix A).

Furthermore, the comparison of CME with SME fractions showed that Ace, Mep1b and Ctsa (ECM regulators, and matrisome-associated proteins) were significantly higher in SME-F1 fraction, compared to CME, whereas CME obtained more abundances of core matrisome proteins including ECM glycoproteins such as Nid1, Nid2, Tinagl1 and Agrn. There were significant FC differences for some matrisome proteins in the comparison between SME-F2 fraction and CME. Serpina1a and A2m are matrisome-associated ECM regulators and were 49.8 (*p*-value < 0.0001) and 20.1 (*p*-value < 0.001) folds higher in SME-F2, respectively. Nid1 and Lamb1 are core matrisome ECM glycoproteins and were 64.4 (*p*-value < 0.001) and 33.3 (*p*-value < 0.001) folds higher in CME, respectively, compared to SME-F2. These observations indicate that specific extraction methods may be required for the enrichment of specific matrisome proteins. The SME-F3 and CME comparison also showed that the majority of highly abundant proteins are matrisome-associated (e.g., ECM-affiliated proteins and ECM regulators), whereas core matrisome proteins (e.g., ECM glycoproteins and collagens) were higher in the CME.

**Figure A4:**
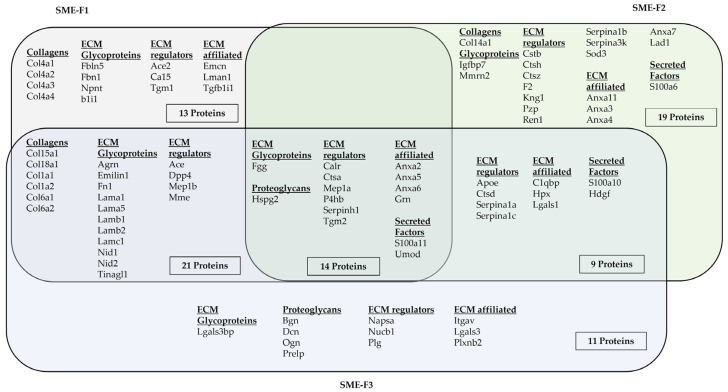
Quantified matrisome proteins in fractions of SME. Venn diagram illustrates the common and unique matrisome proteins in each fraction. Matrisome proteins were classified as collagens, ECM glycoproteins, proteoglycans, ECM-affiliated, ECM regulators and secreted factors.

In addition, we compared the composition of the quantifiable matrisome proteins in SME fractions (Figure A4 in Appendix A). This analysis showed that 48, 42 and 56 matrisome proteins were quantitatively detected in SME-F1, SME-F2, and SME-F3, respectively. Among core matrisome proteins, collagens and glycoproteins were mostly detected (LFQ intensities) in SME-F1 and SME-F3, while proteoglycans were predominantly detected in SME-F3. In parallel, different ECM-associated proteins were detected in different fractions. However, SME-F2 allowed for the detection of more ECM regulators, ECM-affiliated proteins and secreted factors alone compared to other fractions.

## 3. Discussion

The current understanding of matrisome composition and how it is regulated during pathophysiological processes remains very limited. One of the major obstacles is the need for optimization of ECM protein extraction and characterization methods. Many previous studies which examined kidney tissues of different species mainly used the decellularization approach that was developed for tissue engineering applications and involved removal of cellular proteins followed by matrisome protein examination in the decellularized scaffolds [33,34,35]. However, long detergent incubations in tissue engineering-focused decellularization methods may cause degradation and elimination of some matrisome proteins [36].

In the current study, we aimed to comprehensively characterize the mouse renal matrisome using proteomic technologies. In order to overcome the limitations imposed by the tissue engineering decellularization technologies, we first examined two ECM extraction and enrichment methods (CME and SME) in healthy mouse kidney tissues (see Figure 1).

The first method, CME, relied on using a commercially available Millipore Compartment Protein fractionation kit. This method biochemically separates subcellular proteins and enriches matrisome proteins in the insoluble pellet at the final step of the extraction series. The method is well documented in previous studies, and many ECM proteins have been identified by using this extraction method. For example, Naba et al. [5] identified 100 matrisome proteins in mouse lungs and colon, Schiller et al. [18] identified 435 matrisome proteins in healthy mouse lungs and Gocheva et al. [19] identified 113 matrisome proteins in human lungs. Just recently, this method has been applied for the first time by Lipp et al. [28] to identify 79 matrisome proteins of mouse kidneys. In our study using Millipore Compartment Fractionation (here termed the CME method), we detected 83 matrisome proteins of which 16 proteins were not previously listed in the MD. Although our study and Lipp et al. [28] used the same matrisome enrichment method, the protein precipitation, deglycosylation and LC-MS/MS set-up were different. This could explain the higher total number of matrisome proteins identified in our work, compared to the reference [28]. It is important to note that the Millipore Compartment Fractionation only analyses one fraction, while the loosely bound and soluble matrisome proteins potentially remain in the “cellular” fractions that are not used in matrisome studies.

In an attempt to improve the ECM protein isolation on the second half of the same kidney sample, we applied another matrisome enrichment method, a sequential matrix extraction (SME). This method allowed the extraction of loosely bound or soluble matrisome proteins using a high-salt buffer before removal of cellular components by SDS and enriched the matrisome fractions by solubilizing the insoluble pellet with Gu-HCl [22]. Using a method similar to our SME approach, Massey et al. [23] identified 79 matrisome proteins from all three fractions of mouse liver. This extraction technique is potentially more comprehensive for identifying matrisome proteins, but it has not yet been optimized for kidney tissue. Our study, for the first time, used this sequential approach (SME) to identify matrisome proteins in mouse kidneys. We have identified 105 matrisome proteins, of which 22 proteins were yet to be listed in the currently available MD (http://matrisomeproject.mit.edu/, accessed on 23 January 2023) (see Figure 2). Compared to the results obtained by Lipp et al. [28], our study could distinctly detect 57 matrisome proteins. These 57 proteins included mostly the matrisome division of ECM-associated proteins. We can infer from these findings that SME fractions allow for more efficient detection of ECM-associated proteins compared to CME.

It should be noted that the location of most of the 22 proteins have not previously been exclusively attributed to ECM compartments, but to different “Gene Ontology-Cellular Components” or/and “Subcellular Locations”, including ECM space/regions in UniProt. For example, Nucleobindin-1 (Nucb1) was previously shown to be localized in the Golgi apparatus, cytoplasm, or extracellular regions which were inferred from genome-based computational annotations (https://www.uniprot.org/help/gene_ontology). However, it was additionally manually asserted in UniProt as “Secreted” and recently it has been shown that Nucb1 is secreted with metalloproteinase-2 (MMP-2) and regulates ECM remodeling [37]. Hence, we described this protein as an ECM-regulator. Another set of examples includes enzymes with transmembrane domains such as Ace, Ace2, Dpp-4 and Mme. These enzymes are localized at the cell surface and have extracellular, cytoplasmic and transmembrane domains. However, it has been shown that they may also be present in soluble forms after cleavage and play a role in ECM remodeling ([37,38,39,40,41,42]). Similar to the examples above, we referred to the literature in order to confirm the identified proteins as components of the matrisome (see Table A7 in Appendix A).

We then compared mouse matrisome proteins identified by studies of McCabe et al. [20], Lipp et al. [28] and Liu et al. [30], and human matrisome proteins identified by the studies of Louzao-Martinez et al. [31] and Randles et al. [32]. These studies also differed in matrisome protein enrichment, protein precipitation, deglycosylation, digestion and LC-MS/MS methods (see Figure A1, Table A9 in Appendix A). The comparison was undertaken to identify mouse and human species-specificity of kidney matrisome proteins. The comparison could identify 95 matrisome proteins only identified in mice and 44 only identified in human kidneys (see Table A10, Table A11, Table A12, Table A13, Table A14 and Table A15 in Appendix A). Interestingly, five of the matrisome proteins shown in this study—namely Lgals3bp (glycoprotein), Mfge8 (glycoprotein) Emcn (ECM-affiliated), Apoe (ECM regulator) and Sod3 (ECM regulator)—were previously identified only in human kidneys but not in mouse kidneys [32]. However, among mouse kidney studies, only our work identified those proteins in mouse kidneys. Hence, our study shows the necessity of using different approaches to identify matrisome proteins and thus develop a robust matrisome protein list.

In addition to matrisome protein identification, our study for the first time allowed quantification of the ECM proteins extracted from heathy mouse kidneys. We performed a discovery proteomics study known as shotgun proteomics, which uses bottom-up approaches based on unbiased analysis. To compare the quantities of proteins obtained via each extraction method, we applied label-free-based quantification (LFQ) data generated with MaxQuant analysis [29]. This is a widely used method in which the quantitation can be performed either based on chromatographic ion intensities or based on spectral counting of identified proteins [43]. It provides high throughput and does not have the limitations of label-based quantifications such as high complexity of sample preparation, requirement of high concentrations of samples and incomplete labelling [15,43]. In our study using LFQ, we successfully quantified 57 matrisome proteins via CME and 81 proteins via SME (see Figure 5). Some of the quantifiable proteins were more abundant either in CME or SME. This study demonstrated that CME could detect greater intensities of core matrisome proteins, but fewer ECM-associated proteins, compared to SME (see Figure 4). To show the benefit of each extraction method, which can be used as a guideline to study different matrisome proteins in different kidney diseases, we compared SME fractions with CME, separately (see Table S2 in Supplementary Information). For example, IgA nephropathy (IgAN), which is one of the most prevalent chronic glomerular diseases, had significantly more matrisome proteins (Col4a1, Lamb1, Hspg2, Emilin, Fgg and Fbln) in diseased patients compared to healthy patients [34]. These proteins could be better detected using CME, while Col4a1 and Col15a1, which can be detected by both methods and were also elevated in IgAN, could be better detected by CME or in the SME-F3 fraction. In renal fibrosis, the hallmark of CKD, it is known that Col 1, 2, 3, 5, 6, 7 and 15, Fn1, Dcn and Bgn accumulate in the ECM [44]. We detected those matrisome proteins using both extraction methods, although some of them had higher abundances in CME product. Collectively, CME was powerful at extracting several matrisome proteins in higher abundances, while SME allowed for the quantification of more matrisome proteins.

Subsequently, we quantitatively analyzed matrisome proteins in SME fractions. It has previously been shown in mouse lung [21] and liver [23] that sequential extraction methods, similar to that of the SME used in our study, can extract loosely bound matrisome proteins by using a NaCl buffer which displaces polyanionic interactions and further solubilizes the insoluble proteins using Gu-HCl [21,23]. The SME-F1 fraction allowed detection of mostly secreted, basement membrane proteins and some interstitial matrix proteins (Col1 and Col6). SME-F3 distinctly revealed proteoglycans which could not be found in other fractions, but also detected interstitial matrix and basement membrane proteins. Conversely, the SME-F2 fraction allowed for detection of only a few core matrisome proteins which are located in the interstitial matrix, and also secreted and basement membrane proteins. Although the fractions of the extracted samples revealed matrisome proteins from different ECM locations, each fraction had advantages in extracting different matrisome proteins.

Although many matrisome proteins identified in this study have also been previously identified in other studies, our study was able to detect additional matrisome proteins. The most plausible explanation is the use of different extraction methods. For example, LOX enzymes, which are involved in collagen cross-linking, could not be detected in our extraction methods, but were detected in another mouse kidney proteomic study where different extraction methods were used with a combination of a modified deglycosylation/protein digestion process [20,28]. In addition, the removal of glycosaminoglycans (GAGs) by digestion enzymes such as chondroitinase and heparinase has been suggested to improve peptide identification [22,45,46]. However, McCabe et al. [20] reported that using GAG-digesting enzymes only improved proteoglycan identification, but not for other matrisome proteins. It is important to note that this study was performed on healthy kidney tissue, while it is known that GAG deposition in the ECM is increased during disease, e.g., fibrosis [47,48]. Hence, the opportunities for the improvement of analysis by GAG-digesting enzymes are still not completely clear, and our study is limited as we used only PNGaseF to remove N-glycans. We would suggest performing a pilot study using GAG-digesting enzymes to analyze whether it improves matrisome protein identification before applying it to all samples. Finally, the native biological variation in the proteome needs to be considered. In particular, the role of the age of the animals has been demonstrated in several tissue-specific proteomic studies in mice [49,50].

## 4. Materials and Methods

### 4.1. Animal Preparation and Kidney Tissue Collection

Mouse kidney tissues were obtained via the post-mortem animal tissues sharing program encouraged and approved by the UNSW Animal Care and Ethics Committee (ACEC). WT C57BL/6 mice (female, aged 4–10 weeks, Australian Bioresources, Moss Vale NSW, Australia) were housed in a stable environment at 21 ± 2 °C with a 12 h/12 h light-dark cycle. On the day of experimentation, the animals were anesthetized with 4% vaporized isoflurane delivered into an induction chamber and euthanized by cervical dislocation. First, the retinas were collected for the main experiment approved by the UNSW ACEC. After that, the animal bodies were placed on ice and underwent further dissection and kidney extirpation. The obtained kidneys were kept on ice, and one of the kidneys per animal was quickly transversely cut. Then, each half of a kidney (~50 mg) per animal was designated for CME and SME protein extraction (see Figure 1a).

### 4.2. ECM Proteins Extraction

The dissected kidney tissue samples were processed using the CME and SME methods of protein extraction as described below. For each extraction method, three biological replicates were used.

#### 4.2.1. Compartmental Matrix Enrichment (CME) Method

The dissected kidney halves were grinded using a glass tissue homogenizer, on ice. The homogenized tissue was processed using the CME method in the following way. The Millipore Compartment Protein Extraction Kit (Merck-Millipore, Bayswater VIC 3153, Australia, Cat. #2145) was used to deplete cytosolic, nuclear, membrane and cytoskeletal proteins and to enrich ECM proteins as described in [17]. The obtained ECM enriched pellets were washed with 1× PBS (1.7 mM KH_2_PO_4_, 5 mM Na_2_HPO_4_, 150 mM NaCl, 25 mM EDTA, pH 7.4) containing 1:100 (*v*:*v*) of protease inhibitors (PI) (Halt Protease Inhibitor Cocktail, #78429, Thermo Scientific, North Ryde NSW 2113, Australia) three times to remove detergents, and stored at −20 °C.

#### 4.2.2. Sequential Matrix Enrichment (SME) Method

When using the SME method, enrichment of ECM proteins was performed according to the protocol described elsewhere [22]. Briefly, half-kidney samples were diced into 2–3 mm pieces and washed five times with ice-cold 1x PBS containing 1:100 (*v*:*v*) of PI (Halt Protease Inhibitor Cocktail, #78429, Thermo Scientific) to minimize blood contamination. To extract ECM-associated, loosely bound ECM proteins and newly synthesized ECM proteins, the washed samples were incubated in NaCl buffer (0.5 M NaCl, 10 mM Tris-HCl and 25 mM EDTA, pH 7.5 and 1:100 (*v*:*v*) of PI) for 1 h at room temperature (RT) by vortexing at a speed of 65 rpm (Stuart Orbital Shaker). Samples were centrifuged at 16,000× *g* for 10 min at 4 °C and the supernatant was saved as Fraction 1. The pellet was treated with SDS buffer (0.1% SDS, 25 mM EDTA and 1:100 (*v*:*v*) of PI) by vortexing at RT for 16 h at a speed of 65 rpm and was centrifuged at 16,000× *g* for 10 min at 4 °C to separate intracellular proteins. Then, pellets were treated with GuHCl buffer (4 M guanidine hydrochloride, 50 mM Na acetate, 25 mM EDTA, pH 5.8, and 1:100 (*v*:*v*) of PI) by vortexing at RT for 72 h at speed 225 rpm to solubilize ECM proteins. Supernatants after centrifugation at 16,000 × *g* for 10 min at 4 °C were saved as Fraction 2 and pellets were saved as Fraction 3 after washing three times with ice-cold 1x PBS containing 1:100 (*v*:*v*) of PI. All saved fractions were stored at −20 °C.

#### 4.2.3. Protein Precipitation

Pellets obtained from the CME and the three fractions obtained via SME methods (see Figure 1b) were precipitated in EtOH separately to remove detergents/agents. Ten times volume of ice-cold 100% EtOH was added to each fraction and incubated overnight at −20 °C. Protein precipitates were obtained by centrifugation at 16,000× *g* for 10 min at 4 °C and then by drying pellets. Dried pellets were resuspended in the buffer (8 M urea in 100 mM ammonium bicarbonate, pH 8.0) for the following downstream processes.

#### 4.2.4. Deglycosylation and In-Solution Digestion

Deglycosylation and in-solution digestion were performed as described in [17]. Briefly, the obtained pellets for each fraction after protein precipitation were resuspended by adding the appropriate volume (50 µL/5–10 mg dry weight) of 8 M urea and dithiotreitol (DTT) at a final concentration of 10 mM. Samples were incubated with continuous agitation at 150 rpm (Stuart Orbital Shaker) for 2 h at 37 °C.

Alkylation was performed by adding iodoacetamide (IAA) to a final concentration of 25 mM. To complete alkylation, the DTT:IAA ratio was adjusted to 1: 2.5 and incubation was carried out in the dark for 30 min at RT.

Deglycosylation was performed by diluting urea to 2M using 100 mM ammonium bicarbonate, pH 8.0 and adding 2 µL/5–10 mg dry weight (DW) of PNGaseF (Peptide-N-Glycosidase F) (New England Biolabs, #P0704S). Samples were incubated with continuous agitation at 150 rpm (Stuart Horizontal Shaker) for 2 h at 37 °C.

Digestion was continued by diluting samples with 100 mM ammonium bicarbonate pH 8.0 to reduce the concentration of urea in the samples’ solution to 1 M. Then, 2 µL/5–10 mg DW of Trypsin/Lys-C (Endoproteinase LysC) Mix (Promega, Cat #V5071) was added to samples and incubated with continuous agitation at 150 rpm overnight at 37 °C. The digestion reaction was inactivated with freshly prepared 8 µL of 50% trifluoro-acetic acid (TFA) per 5–10 mg DW of pellet to reach a final concentration of 1% of TFA. The acidified samples were centrifuged at 16,000× *g* for 5 min at RT and the supernatants were saved for desalting steps.

Desalting was performed by using Pierce C18 stage tips (#SP301, Thermo Scientific). Prior to proteomics analysis, desalted peptides were eluted with freshly prepared HPLC-grade water-based solution containing 60% acetonitrile and 0.1% TFA, followed by concentrating in a vacuum concentrator. Samples were then resuspended in freshly prepared 3% acetonitrile and 0.1% TFA water-based solution and analyzed via LC-MS/MS.

### 4.3. LC-MS/MS and Data Analysis

Digested peptides were separated using nanoLC with an Ultimate nanoRSLC UPLC and autosampler system (Dionex, Amsterdam, The Netherlands). Samples (2.5 µL) were concentrated and desalted onto a micro C18 precolumn (300 µm × 5 mm, Dionex) with H_2_O:CH_3_CN (98:2, 0.1% TFA) at 15 µL/min. After a 4 min wash the pre-column was switched (Valco 10 port UPLC valve, Valco, Houston, TX, USA) into line with a fritless nano-column (75 µ × ~20 cm) containing C18AQ media (1.9 µ, 120 Å Dr Maisch, Ammerbuch-Entringen, Germany) manufactured according to Gatlin. Peptides were eluted using a linear gradient of H_2_O:CH_3_CN (98:2, 0.1% formic acid) to H_2_O:CH_3_CN (64:36, 0.1% formic acid) at 200 nL/min over 30 min. High voltage (2000 V) was applied to low-volume Titanium union (Valco) with the column oven-heated to 45 °C (Sonation, Biberach, Germany) and the tip positioned at ~0.5 cm from the heated capillary (T = 300 °C) of a QExactive Plus (Thermo Electron, Bremen, Germany) mass spectrometer. Positive ions were generated by electrospray and the QExactive operated in data-dependent acquisition mode (DDA).

A survey scan *m*/*z* 350–1750 was acquired (resolution = 70,000 at *m*/*z* 200, with an accumulation target value of 1,000,000 ions) and lock mass enabled (*m*/*z* 445.12003). Up to the 10 most abundant ions (>80,000 counts, underfill ratio 10%) with charge states > +2 and <+7 were sequentially isolated (width *m*/*z* 2.5) and fragmented by higher-energy C-trap dissociation (HCD) (normalized collision energy, NCE = 30) with an automatic gain control (AGC) target of 100,000 ions (resolution = 17,500 at *m*/*z* 200). M/z ratios selected for MS/MS were dynamically excluded for 30 s.

MS raw files were analyzed by the MaxQuant software [51] (version 2.0.3.0), and peak lists were searched against the mouse UniProt FASTA (reviewed and unreviewed) database (version Aug 2019), and a common contaminants database using the Andromeda search engine [52]. For protein identification and LFQ quantification, fractions of SME were nominated as fractions in MaxQuant [53], and a proprietary Maxquant algorithm was applied to merge and normalize the fractions to give an output as one sample. A fixed modification carbamidomethylation (C) and variable modifications, oxidation (M,P), acetyl (protein N-term), hydroxyproline, and deamidation (N,Q) were used. The false discovery rate was set to 1% for proteins and peptides (minimum length of 7 amino acids) and was determined by searching a reverse database. Enzyme specificity was set as trypsin and lys-C, and a maximum of two missed cleavages were allowed in the database search. Peptide identification was performed with an allowed precursor mass deviation of up to 4.5 ppm after time-dependent mass calibration and an allowed fragment mass deviation of 20 ppm. For LFQ, in MaxQuant, the minimum ratio count was set to two. For matching between runs, the retention time alignment window was set to 30 min and the match time window was 1 min.

For protein identification in CME vs. SME—the first round of matrisome protein identification—unique peptides which were present in at least two of the biological replicates were searched using “Matrisome Annotator” in the MD (http://matrisomeproject.mit.edu/, accessed on 23 January 2023). The matrisome proteins were classified as core matrisome proteins (collagens, ECM glycoproteins, and proteoglycans) and matrisome-associated proteins (ECM-affiliated, ECM Regulators, and Secreted Factors). In the second round, the protein list obtained from LC-MS/MS was matched using the UniProt Database for *Mus musculus*, and possible matrisome proteins were searched based on their tissue locations using “extracellular matrix”, “extracellular space”, “basement membrane”, “secreted”, “lysosome” and “exosome”. To verify potential candidates, their possible interactions with matrisome proteins were searched in protein interaction databases (BioGRID, STRING). In the final stage, the short list of proteins was searched in the literature to confirm that proteins have been located in the ECM by other studies.

For protein quantification, LFQ intensity values were taken from the MaxQuant protein “Groups” table, which represented the values after inter-experiment normalization [54]. Differential abundant analysis was performed using the LFQ-Analyst online software (https://bioinformatics.erc.monash.edu/apps/LFQ-Analyst/, accessed on 23 January 2023) [29]. Matrisome proteins which had LFQ intensities in at least 2 of 3 biological replicates were included in the comparison of CME and SME or SME-fractions. In LFQ-Analyst, parameters were set as Perseus-type imputation, adjusted *p*-value cutoff <0.05 and Log2 fold- change (FC) cutoff = 1 (i.e., 2-FC). The comparison was presented with FC and *p*-values. All *p*-values were corrected for multiple hypothesis testing using the Benjamini–Hochberg method. Proteins with *p* < 0.05 and up- or downregulated 2-FC were presented as significantly different between the samples obtained by the extraction methods CME and SME.

To compare the fractions of the SME and CME products, LFQ analysis was performed separately for each SME fraction, and the same parameters used to compare CME and SME were applied, although in this fraction comparison analysis, fractions were set as “separate method” instead of as “fractions”.

## 5. Conclusions

In conclusion, a better understanding of the mouse kidney matrisome is likely to inform future studies in the areas of kidney disease modelling, kidney development biology, ageing and tissue engineering. In this study, we have successfully progressed knowledge towards understanding the composition of the mouse kidney matrisome via identification and quantification of matrisome proteins and via identification of a set of proteins which were not previously listed as matrisome components. In addition, this study showed the importance of using different protein extraction steps to fractionate matrisome proteins for a better understanding of the mouse kidney matrisome composition. Hence, we suggest to obtain different fractions of matrisome proteins to discover more ECM proteins for further studies of kidney ECM turnover in normal and pathological conditions, and also to identify novel drug targets and biomarkers for the treatment and diagnosis of chronic kidney disease.

## Data Availability

The data presented and discussed in this study is available in (Appendix A and Supplementary Material Tables S1 and S2).

## Supplementary Materials

The following supporting information can be downloaded at: https://www.mdpi.com/article/10.3390/ijms24032827/s1.

## Appendix A

**Table A1:** The list of core matrisome proteins belonging to the category of collagens and identified in at least two of the biological replicates.

Protein ID	Protein Name	Gene Symbol	Identified by Method		Ref *
			CME	SME	
P11087	Collagen alpha-1(I) chain	Col1a1	+	+	MD
Q01149	Collagen alpha-2(I) chain	Col1a2	+	+	MD
P08121	Collagen alpha-1(III) chain	Col3a1	+	+	MD
P02463	Collagen alpha-1(IV) chain	Col4a1	+	+	MD
P08122	Collagen alpha-2(IV) chain	Col4a2	+	+	MD
Q9QZS0	Collagen alpha-3(IV) chain	Col4a3	+	−	MD
Q9QZR9	Collagen alpha-4(IV) chain	Col4a4	+	−	MD
Q9CPW5	Collagen alpha-5(IV) chain	Col4a5	+	+	MD
O88207	Collagen alpha-1(V) chain	Col5a1	+	+	MD
Q3U962	Collagen alpha-2(V) chain	Col5a2	+	+	MD
Q04857	Collagen alpha-1(VI) chain	Col6a1	+	+	MD
Q02788	Collagen alpha-2(VI) chain	Col6a2	+	+	MD
Q61001	Collagen alpha-3(VI) chain	Col6a3	+	+	MD
Q80X19	Collagen alpha-1(XIV) chain	Col14a1	+	+	MD
O35206	Collagen alpha-1(XV) chain	Col15a1	+	+	MD
P39061	Collagen alpha-1(XVIII) chain	Col18a1	+	+	MD

Abbreviations: CME—compartmental matrix enrichment; SME—sequential matrix enrichment; MD—Matrisome Database; Reference *—source of information for the provided protein identification; “+” indicates that protein has been identified; “−“ indicates that protein has not been identified by the method.

**Table A2:** The list of core matrisome proteins belonging to the category of glycoproteins and identified in at least two of the biological replicates. A row highlighted in yellow describes an identified matrisome protein which was not previously listed in the MD.

Protein ID	Protein Name	Gene Symbol	Identified by Method		Ref *
			CME	SME	
A2ASQ1	Agrin	Agrn	+	+	MD
Q9QZZ6	Dermatopontin	Dpt	+	−	MD
Q91VF5	EMI domain-containing protein 1	Emid1	+	+	MD
Q99K41	EMILIN-1	Emilin1	+	+	MD
Q61554	Fibrillin-1	Fbn1	+	−	MD
Q61555	Fibrillin-2	Fbn2	−	+	MD
E9PV24	Fibrinogen alpha chain	Fga	+	+	MD
Q8VCM7	Fibrinogen gamma chain	Fgg	+	+	MD
P11276	Fibronectin	Fn1	+	+	MD
A2ASQ1	Progranulin	Grn	−	+	MD
Q61581	Insulin-like growth factor-binding protein 7	Igfbp7	+	+	MD
P19137	Laminin subunit alpha-1	Lama1	+	+	MD
P97927	Laminin subunit alpha-4	Lama4	+	+	MD
Q61001	Laminin subunit alpha-5	Lama5	+	+	MD
P02469	Laminin subunit beta-1	Lamb1	+	+	MD
Q61292	Laminin subunit beta-2	Lamb2	+	+	MD
P02468	Laminin subunit gamma-1	Lamc1	+	+	MD
Q07797	Galectin-3-binding protein	Lgals3bp	+	+	[55]
Q8K4G1	Latent TGF-b-binding protein 4	Ltbp4	−	+	MD
P21956	Lactadherin	Mfge8	+	+	MD
A6H6E2	Multimerin-2	Mmrn2	+	+	MD
P10493	Nidogen-1	Nid1	+	+	MD
O88322	Nidogen-2	Nid2	+	+	MD
Q91V88	Nephronectin	Npnt	−	+	MD
Q99JR5	Tubulointerstitial nephritis antigen-like	Tinagl1	+	+	MD
P29788	Vitronectin	Vtn	+	−	MD
Q8R2Z5	von Willebrand factor A domain-containing protein 1	Vwa1	+	−	MD
Q99KC8	von Willebrand factor A domain-containing protein 5A	Vwa5a	−	+	MD

Abbreviations: CME—compartmental matrix enrichment; SME—sequential matrix enrichment; MD—Matrisome Database; Ref *—source of information for the provided protein identification; ; “+” indicates that protein has been identified; “−“ indicates that protein has not been identified by the method.

**Table A3:** The list of core matrisome proteins belonging to the category of proteoglycans and identified in at least two of the biological replicates.

Protein ID	Protein Name	Gene Symbol	Identified by Method		Ref *
			CME	SME	
Q99MQ4	Asporin	Aspn	+	−	MD
P28653	Biglycan	Bgn	+	+	MD
P28654	Decorin	Dcn	+	+	MD
Q05793	Heparan sulphate proteoglycan core protein	Hspg2	+	+	MD
P51885	Lumican	Lum	+	+	MD
Q62000	Mimecan	Ogn	+	−	MD
Q9JK53	Prolargin	Prelp	+	+	MD

Abbreviations: CME—compartmental matrix enrichment; SME—sequential matrix enrichment; MD—Matrisome Database; Ref *—source of information for the provided protein identification; ; “+” indicates that protein has been identified; “−“ indicates that protein has not been identified by the method.

**Table A4:** The list of matrisome-associated proteins belonging to the category of ECM regulators and identified in at least two of the biological replicates. Rows highlighted in yellow describe identified matrisome proteins which were not previously listed in the MD.

Protein ID	Protein Name	Gene Symbol	Identified by Method		Ref *
			CME	SME	
Q8JZZ0	Alpha-2-macroglobulin; Alpha-2-macroglobulin 165 kDa subunit; Alpha-2-macroglobulin 35 kDa subunit	A2m	−	+	MD
P09470	Angiotensin-converting enzyme	Ace	+	+	[40]
Q8R0I0	Angiotensin-converting enzyme 2	Ace2	+	+	[38,39]
O35598	Disintegrin and metalloproteinase domain-containing protein 10	Adam10	+	+	MD
P08226	Apolipoprotein E	Apoe	−	+	[56]
P50429	Arylsulfatase B	Arsb	−	+	[57]
Q99N23	Carbonic anhydrase 15	Ca15	+	+	[58]
Q62426	Cystatin-B	Cstb	-	+	MD
P16675	Lysosomal protective protein	Ctsa	+	+	MD
P10605	Cathepsin B	Ctsb	+	+	MD
P18242	Cathepsin D	Ctsd	+	+	MD
P49935	Pro-cathepsin H	Ctsh	+	+	MD
Q9WUU7	Cathepsin Z	Ctsz	−	+	MD
P28843	Dipeptidyl peptidase 4	Dpp4	+	+	[59,60]
P19221	Prothrombin	F2	−	+	MD
Q571E4	N-acetylgalactosamine-6-sulfatase	Galns	−	+	[61]
Q8BFR4	N-acetylglucosamine-6-sulfatase	Gns	−	+	[62]
Q9ESB3	Histidine-rich glycoprotein	Hrg	−	+	MD
Q61703	Inter-alpha-trypsin inhibitor heavy chain H2	Itih2	−	+	MD
O08677	Kininogen-1	Kng1	−	+	MD
P28825	Meprin A subunit alpha	Mep1a	+	+	MD
Q61847	Meprin A subunit beta	Mep1b	+	+	MD
Q61391	Neprilysin	Mme	+	+	[42]
Q02819	Nucleobindin-1	Nucb1	+	+	[37]
P09103	Protein disulfide-isomerase	P4hb	+	+	[63,64]
P20918	Plasminogen	Plg	+	+	MD
P06281	Renin-1	Ren1	−	+	[65]
P07758	Alpha-1-antitrypsin 1-1	Serpina1a	−	+	MD
P22599	Alpha-1-antitrypsin 1-2	Serpina1b	−	+	MD
Q00896	Alpha-1-antitrypsin 1-3	Serpina1c	−	+	MD
P07759	Serine protease inhibitor A3K	Serpina3k	−	+	MD
Q60854	Serpin B6	Serpinb6	−	+	[66]
P19324	Serpin H1	Serpinh1	+	+	MD
O09164	Extracellular superoxide dismutase	Sod3	+	+	[67]
Q9JLF6	Protein-glutamine gamma-glutamyltransferase K	Tgm1	+	+	MD
P21981	Protein-glutamine gamma-glutamyltransferase 2	Tgm2	+	+	MD

Abbreviations: CME—compartmental matrix enrichment; SME—sequential matrix enrichment; MD—Matrisome Database; Ref *—source of information for the provided protein identification; ; “+” indicates that protein has been identified; “−“ indicates that protein has not been identified by the method.

**Table A5:** The list of matrisome-associated proteins belonging to the category of ECM-affiliated proteins and identified in at least two of the biological replicates. Rows highlighted in yellow describe identified matrisome proteins which were not previously listed in the MD.

Protein ID	Protein Name	Gene Symbol	Identified by Method		Ref *
			CME	SME	
P10107	Annexin A1	Anxa1	−	+	MD
P07356	Annexin A2	Anxa2	+	+	MD
O35639	Annexin A3	Anxa3	−	+	MD
P97429	Annexin A4	Anxa4	−	+	MD
P48036	Annexin A5	Anxa5	+	+	MD
P14824	Annexin A6	Anxa6	+	+	MD
Q07076	Annexin A7	Anxa7	−	+	MD
P97384	Annexin A11	Anxa11	−	+	MD
O35658	Complement component 1 Q subcomponent-binding protein	C1qbp	+	+	[68,69]
Q62165	Dystroglycan	Dag1	+	+	[70]
Q9R0H2	Endomucin	Emcn	+	+	MD
Q91X72	Hemopexin	Hpx	−	+	MD
P57016	Ladinin-1	Lad1	+	+	[71,72]
P16045	Galectin-1	Lgals1	+	+	MD
P16110	Galectin-3	Lgals3	+	+	MD
Q9D0F3	Protein ERGIC-53	Lman1	+	+	MD
P11152	Lipoprotein lipase	Lpl	+	+	[73,74]
B2RXS4	Plexin-B2	Plxnb2	+	+	MD
Q62219	TGF-b-1-induced transcript 1 protein	Tgfb1i1	+	+	[75]

Abbreviations: CME—compartmental matrix enrichment; SME—sequential matrix enrichment; MD—Matrisome Database; Ref *—source of information for the provided protein identification; ; “+” indicates that protein has been identified; “−“ indicates that protein has not been identified by the method.

**Table A6:** The list of matrisome-associated proteins belonging to the category of secreted factors and identified in at least two of the biological replicates. Rows highlighted in yellow describe identified matrisome proteins which were not previously listed in the MD.

Protein ID	Protein Name	Gene Symbol	Identified by Method		Ref *
			CME	SME	
P01132	Pro-epidermal growth factor	Egf	−	+	MD
Q8CJ70	Interleukin-19	Il19	+	+	MD
P14069	Protein S100-A6	S100a6	−	+	MD
P08207	Protein S100-A10	S100a10	+	+	MD
P50543	Protein S100-A11	S100a11	+	+	MD
Q91X17	Uromodulin	Umod	+	+	[76,77]
P51859	Hepatoma-derived growth factor	Hdgf	+	+	[78,79]

Abbreviations: CME—compartmental matrix enrichment; SME—sequential matrix enrichment; MD—Matrisome Database; Ref *—source of information for the provided protein identification; ; “+” indicates that protein has been identified; “−“ indicates that protein has not been identified by the method.

**Table A7:** Summary of the newly detected and classified mouse kidney matrisome proteins that are not listed in the current Matrisome Database.

Protein ID	Protein Name	Gene Symbol	Identified by Methods		Role of Protein	Reference
			CME	SME		
***Core matrisome: ECM Glycoproteins***						
Q07797	Galectin-3-binding protein	Lgals3bp	+	+	ECM modulation, immune response	[55]
***Matrisome-associated proteins: ECM Regulators***						
P09470	Angiotensin-converting enzyme	Ace	+	+	ECM remodeling	[40]
Q8R0I0	Angiotensin-converting enzyme 2	Ace2	+	+	ECM remodeling	[38,39]
P08226	Apolipoprotein E	Apoe	−	+	Protein binding	[56]
P50429	Arylsulfatase B	Arsb	−	+	Modulation of signaling in cytoskeletal rearrangement and a component of ECM	[57]
Q99N23	Carbonic anhydrase 15	Ca15	+	+	Regulation of acid-base balance	[58]
P28843	Dipeptidyl peptidase 4	Dpp4	+	+	Signaling and endothelial-mesenchymal transition	[59,60]
Q571E4	N-acetylgalactosamine-6-sulfatase	Galns	−	+	Glycosaminoglycan degradation	[61]
Q8BFR4	N-acetylglucosamine-6-sulfatase	Gns	−	+	Glycosaminoglycan degradation	[62]
Q61391	Neprilysin	Mme	+	+	Elastin degradation	[42]
Q02819	Nucleobindin-1	Nucb1	+	+	ECM remodeling and metalloptroteinase-2 transport	[37]
P09103	Protein disulfide-isomerase	P4hb	+	+	Metalloproteinase activation and platelet adhesion and migration	[63,64]
P06281	Renin-1	Ren1	−	+	Protease activity	[65]
Q60854	Serpin B6	Serpinb6	−	+	Protease binding	[66]
O09164	Extracellular superoxide dismutase	Sod3	+	+	Removal of oxygen radicals and response to hypoxia	[67,80]
***Matrisome-associated proteins: ECM-affiliated Proteins***						
O35658	Complement component 1 Q subcomponent-binding protein	C1qbp	+	+	Regulation of complement activation and cell adhesion	[68,69]
Q62165	Dystroglycan (α-Dystroglycan)	Dag1	+	+	Laminin and dystroglycan binding	[70,81]
P57016	Ladinin-1	Lad1	+	+	Component of basement membrane and integrin signaling	[71,72]
P11152	Lipoprotein lipase	Lpl	+	+	Heparan sulphate binding and lipolytic processing	[73,74]
Q62219	TGF-b-1-induced transcript 1 protein	Tgfb1i1	+	+	ECM remodeling and deposition	[75]
***Matrisome-associated proteins: Secreted Factors***						
Q91X17	Uromodulin	Umod	+	+	Regulation of kidney electrolyte balance	[76,77]
P51859	Hepatoma-derived growth factor	Hdgf	+	+	Matrix bound growth factor	[78,79]

Abbreviations: CME—compartmental matrix enrichment; SME—sequential matrix enrichment; “+” indicates that protein has been identified; “−“ indicates that protein has not been identified by the method.

**Table A8:** Composition of mouse kidney matrisome proteins identified in this study depending on the ECM enrichment method.

Categories of Identified Proteins	Number of Identified Proteins		
	CME	SME	Total, CEM and SME *
Total proteins	1526	2246	2442
Matrisome proteins	83	105	113
*Including:*			
Core matrisome, overall	46	43	51
*Including:*			
*Collagens*	*16*	*14*	*16*
*ECM glycoproteins*	*23*	*24*	*28*
*Proteoglycans*	*7*	*5*	*7*
Matrisome-associated proteins, overall	37	62	62
*Including:*			
*ECM Regulators*	*19*	*36*	*36*
*ECM-affiliated Proteins*	*13*	*19*	*19*
*Secreted Factors*	*5*	*7*	*7*

* This column shows all different proteins (identified based on unique peptides) obtained by CME and SME methods together, without duplication of the proteins found by both methods.

**Table A9:** The comparative analysis of the matrisome composition revealed in mouse and human kidney samples by different studies, including the current study, as well as publications by McCabe et al. [20], Lipp et al. [28], Liu et al. [30], Louzao-Martinez et al. [31], and Randles et al. [32].

Characteristics	Identification of Kidney Matrisome Proteins					
Organism	Mouse	Mouse	Mouse	Mouse	Human	Human
Data source	Our data	McCabe et al.	Lipp et al.	Liu et al.	Louzao-Martinez 2019	Randles 2021
# of methods used	2	4	1	1	1	1
Age	4–10 weeks	N/A	adult	15 weeks	55 years	15–37 years
Gender	F	M	N/A	N/A	M/F	M
Strain	C57BL/6J	C67BL/6J	C57BL/6J	C57BL/6J	N/A	N/A
Size	3	3	3	N/A	13	3
# Total proteins	2442	N/A	N/A	5044	N/A	N/A
# Total matrisomeproteins	113	173	79	139	76	172
# Collagens	16	22	18	16	12	21
# GPs	28	55	38	41	33	53
# PGs	7	10	6	6	6	11
# ECM-regulators	36	57	9	46	11	47
# ECM-affiliated	19	22	6	20	9	28
# Secreted factors	7	7	2	10	5	12

N/A—not available; F—female; M—male; #—number of; GPs—ECM Glycoproteins; PGs—Proteoglycans.

**Table A10:** The combined list of core matrisome proteins belonging to the category of collagens and identified in mouse and human kidneys. The mouse matrisome proteins were obtained from our data and extracted from references [20,28,30]. Human matrisome proteins were obtained from the studies [31,32]. Highlighted “X” refers to identified protein in the corresponding study.

Protein Name	Our data	McCabe 2021	Lipp 2021	Liu 2020	Louzao-Martinez 2019	Randles 2021
	Mouse	Mouse	Mouse	Mouse	Human	Human
COL1A1	X	X	X	X	X	X
COL1A2	X	X	X	X	X	X
COL2A1		X				
COL3A1	X	X	X		X	X
COL4A1	X	X	X	X	X	X
COL4A2	X	X	X	X	X	X
COL4A3	X	X	X	X		X
COL4A4	X	X	X	X		X
COL4A5	X		X	X		X
COL4A6			X			X
COL5A1	X	X	X			X
COL5A2	X	X	X			X
COL6A1	X	X	X	X	X	X
COL6A2	X	X	X	X	X	X
COL6A3	X		X	X	X	X
COL6A5		X		X		
COL6A6		X		X		
COL7A1						X
COL8A1						X
COL11A1		X				
COL11A2		X				
COL12A1		X	X	X	X	X
COL14A1	X	X	X	X	X	X
COL15A1	X	X	X	X	X	X
COL16A1		X				X
COL18A1	X	X	X	X	X	X
COL24A1		X				

**Table A11:** The combined list of core matrisome proteins belonging to the category of ECM glycoproteins and identified in mouse and human kidneys. The mouse matrisome proteins were obtained from our data and extracted from references [20,28,30]. Human matrisome proteins were obtained from the studies [31,32]. Highlighted “X” refers to identified protein in the corresponding study.

Protein Name	Our data	McCabe 2021	Lipp 2021	Liu 2020	Louzao-Martinez 2019	Randles 2021
	Mouse	Mouse	Mouse	Mouse	Human	Human
ADIPOQ				X		
AEBP1		X				
AGRN	X	X	X	X	X	X
AHSG						X
APOH						X
CRELD1						X
CRELD2				X		
DPT	X	X	X		X	X
ECM1		X	X	X		X
EFEMP1		X				X
EFEMP2		X				
ELN		X	X		X	X
EMID1	X	X				
EMILIN1	X	X	X	X	X	X
FBLN1		X		X	X	X
FBLN2		X				
FBLN5	X	X	X		X	X
FBN1	X	X	X	X	X	X
FBN2	X				X	
FGA	X	X	X	X	X	X
FGB		X	X	X	X	X
FGG	X	X	X	X	X	X
FGL1						X
FGL2						X
FN1	X	X	X	X	X	X
FRAS1			X	X	X	X
IGFBP7	X	X	X	X	X	X
KCP		X	X	X		X
LAMA1	X	X	X	X	X	X
LAMA2		X	X	X		X
LAMA3	X	X	X	X		X
LAMA4	X	X	X	X	X	X
LAMA5	X	X	X	X	X	X
LAMB1	X	X	X	X	X	X
LAMB2	X	X	X	X	X	X
LAMB3		X				
LAMC1	X	X	X	X	X	X
LAMC3		X		X		
LGALS3BP	X					X
LTBP1		X	X			
LTBP4	X	X		X		X
MATN2		X	X	X		X
MFAP2		X	X		X	
MFAP4		X		X		X
MFAP5		X		X	X	
MFGE8	X					X
MMRN2	X	X	X	X		X
NID1	X	X	X	X	X	X
NID2	X	X	X	X	X	X
NPNT	X	X	X	X	X	
NTN1				X		X
NTN4		X		X		
PAPLN		X		X		X
POSTN	X	X	X	X	X	X
PXDN		X	X	X		X
SBSPON		X				X
SPARC		X				X
TGFBI		X	X	X	X	X
THBS1		X			X	X
THSD4		X				X
TINAG			X		X	X
TINAGL1	X	X	X	X	X	X
TNC		X	X		X	X
TNXB			X			
VTN	X	X	X	X	X	X
VWA1	X	X	X			X
VWA2		X		X		
VWA5A	X	X		X		
VWF		X			X	X

**Table A12:** The combined list of core matrisome proteins belonging to the category of proteoglycans and identified in mouse and human kidneys. The mouse matrisome proteins were obtained from our data and extracted from references [20,28,30]. Human matrisome proteins were obtained from the studies [31,32]. Highlighted “X” refers to identified protein in the corresponding study.

Protein Name	Our data	McCabe 2021	Lipp 2021	Liu 2020	Louzao-Martinez 2019	Randles 2021
	Mouse	Mouse	Mouse	Mouse	Human	Human
ANGPTL2		X				X
ASPN	X	X	X			X
BGN	X	X	X	X	X	X
DCN	X	X	X	X	X	X
HAPLN1		X				
HSPG2	X	X	X	X	X	X
LUM	X	X	X	X	X	X
OGN	X	X		X		X
PRELP	X	X	X	X	X	X
PRG2		X				X
PRG3						X
VCAN		X			X	X

**Table A13:** The combined list of matrisome-associated proteins belonging to the category of ECM regulators and identified in mouse and human kidneys. The mouse matrisome proteins were obtained from our data and extracted from references [20,28,30]. Human matrisome proteins were obtained from the studies [31,32]. Highlighted “X” refers to identified protein in the corresponding study.

Protein Name	Our Data	McCabe 2021	Lipp 2021	Liu 2020	Louzao-Martinez 2019	Randles 2021
	Mouse	Mouse	Mouse	Mouse	Human	Human
A2M	X	X			X	X
ACE	X					
ACE2	X					
ADAM8				X		
ADAM9						X
ADAM10	X	X		X		
ADAM17				X		
ADAMTSL1		X				
ADAMTSL4		X				
ADAMTSL5						X
AGT		X				
AMBP		X		X	X	X
APOE	X					X
ARSB	X					
CA15	X					
CASP14						X
CPN2		X		X		
CST3		X				
CSTB	X	X		X		
CTSA	X	X		X		X
CTSB	X	X		X		X
CTSC		X				X
CTSD	X	X		X	X	X
CTSF		X				
CTSH	X	X		X		X
CTSL		X		X		
CTSS				X		X
CTSZ	X	X		X		X
DPP4	X					
ELANE						X
F13A1		X				
F2	X	X		X		
F3						X
F9		X		X		X
FAM20B	X	X		X		
GALNS	X					
GANAB						X
GNS	X					
HRG	X	X		X	X	X
HTRA1		X				X
ITIH1		X	X	X	X	X
ITIH2	X	X		X	X	
ITIH4		X		X		X
ITIH5		X				X
KNG1	X	X		X		X
LOX		X				
LOXL1		X				
LOXL2			X			
LOXL4		X				
MEP1A	X	X	X	X		
MEP1B	X	X	X	X		
MME	X					
MMP9						X
MUG2		X		X		
NGLY1				X		
NUCB1	X					
OGFOD2				X		
P4HA1		X				X
P4HB	X					
PLAU		X				
PLG	X	X	X	X		X
PLOD1						X
PLOD2						X
PLOD3						X
PLSCR1						X
PRDX1						X
PRTN3						X
REN1	X					
SERPINA1					X	
SERPINA1A	X	X	X	X		
SERPINA1B	X	X		X		
SERPINA1C	X	X		X		
SERPINA1D		X		X		
SERPINA1E		X		X		
SERPINA3					X	
SERPINA3G				X		
SERPINA3K	X	X		X		
SERPINA3M		X		X		
SERPINA3N		X				
SERPINA5						X
SERPINA6		X		X		
SERPINB1A		X		X		
SERPINB6	X			X		
SERPINB12						X
SERPINC1		X		X	X	X
SERPIND1		X		X		X
SERPINF1		X				X
SERPINF2		X		X		X
SERPING1		X		X		X
SERPINH1	X	X	X	X		X
SOD1						X
SOD3	X					X
ST14		X				
TGM1	X	X	X	X		X
TGM2	X	X	X	X	X	X
TGM3						X
TIMP3		X		X	X	X

**Table A14:** The combined list of matrisome-associated proteins belonging to the category of ECM-affiliated proteins and identified in mouse and human kidneys. The mouse matrisome proteins were obtained from our data and extracted from references [20,28,30]. Human matrisome proteins were obtained from the studies [31,32]. Highlighted “X” refers to identified protein in the corresponding study.

Protein Name	Our data	McCabe 2021	Lipp 2021	Liu 2020	Louzao-Martinez 2019	Randles 2021
	Mouse	Mouse	Mouse	Mouse	Human	Human
ANXA1	X	X		X	X	X
ANXA2	X	X	X	X		X
ANXA3	X	X		X		
ANXA4	X	X		X	X	X
ANXA5	X	X		X	X	X
ANXA6	X	X	X	X	X	X
ANXA7	X	X	X	X	X	X
ANXA9		X				
ANXA11	X	X	X	X	X	X
ANXA13				X		X
APCS						X
C1QBP	X					
CCT2						X
CCT6A						X
CD109						X
CSPG4		X		X		X
CXCL14						X
DAG1	X					
EMCN	X					X
FREM1						X
FREM2		X	X	X	X	
GPC4		X		X		
GRN	X					
HPX	X	X		X		X
LAD	X					
LGALS1	X	X		X	X	X
LGALS2						X
LGALS3	X	X				X
LGALS8		X				X
LGALS9		X		X		
LMAN1	X	X	X	X	X	X
LPL	X					
MBL1		X				
MBL2		X				
MGP						X
MUC1						X
PLXNB1						X
PLXNB2	X	X		X		X
PLXND1				X		X
SDC4				X		
SEMA4D		X		X		
TGFB1I1	X					

**Table A15:** The combined list of matrisome-associated proteins belonging to the category of secreted factors and identified in mouse and human kidneys. The mouse matrisome proteins were obtained from our data and extracted from references [20,28,30]. Human matrisome proteins were obtained from the studies [31,32]. Highlighted “X” refers to identified protein in the corresponding study.

Protein Name	Our data	McCabe 2021	Lipp 2021	Liu 2020	Louzao-Martinez 2019	Randles 2021
	Mouse	Mouse	Mouse	Mouse	Human	Human
CLU						X
CRLF3				X		
DCD						X
DEFA1						X
EGF	X	X		X		
EGFL7		X	X			
FGF1				X		
FGF2						X
HCFC1		X				
HDGF	X					
HRNR					X	X
II19	X					
INHBE						X
PF4				X		
S100A1	X			X		
S100A6	X					
S100A7					X	
S100A8				X	X	X
S100A9	X				X	X
S100A10	X	X		X		X
S100A11	X	X	X	X	X	X
S100A13	X					
S100A14						X
S100G		X		X		
SFRP1				X		
UMOD	X					
